# A Case of Congenital Dropsy of the Cranial, Thoracic, and Abdominal Cavities, Complicated with Spina-Bifida, Impeding Labor, and Rendering Perforation of the Head Necessary to Accomplishing Delivery

**Published:** 1849-07

**Authors:** Thos. Lipscomb

**Affiliations:** Shelbyville, Tennessee


Art. IV.—*A Case of Congenital Dropsy of the Cranial, Thoracic, and Abdominal Cavities, complicated with Spina-Bifida, impeding labor, and rendering perforation of the head necessary to accomplishing delivery.* By Dr. Thos. Lipscomb, of Shelbyville, Tennessee.

I was called, on the night of the 12th of April last, to visit Mrs. P., in consultation with my young friend, Dr. R. A. Coldwell. From Dr. Coldwell I learned that some forty-eight hours or more had elapsed since the commencement of labor, and that he had supervised her case in person for the last forty hours. He informed me that her health was good, and that she had given birth to one child previously without extraordinary difficulty. The membranes had given way early in the morning of the day preceding the night of my visit. Dr. C. informed me that the pains had been strong and forcing, or protrusive in their character for some five or six hours, which characteristics were still manifest; that it was a presentation of the head, and that the os uteri was well dilated, but that there had been no advancement of the presenting part for some five hours.

Having received this brief detail, and witnessing the suffering of Mrs. P., and hearing her importunate cries for relief, I proceeded to make an examination, when I found the presentation somewhat embarrassing, as there was a large and fluctuating tumor which had entered the inferior strait, and above it could be felt a hard substance occupying each iliac fossa of the mother. After repeated examinations by myself and Dr. C., which probably occupied half an hour, we were enabled to determine the true character of the case, which was the following, viz: The soft fluctuating tumor was the scalp distended with fluid, and the more firm portions which seemed to occupy the iliac fossa were the parietal protuberances forced very widely apart by the accumulated fluid. After making this decision, (which Dr. C. had suspected before my arrival), and ascertaining that the different sutures were very widely separated, (the sagittal, for instance, two and a half or three inches) we were fully satisfied that nothing less than perforating the head and evacuating the fluid would be availing. This opinion was immediately communicated to the husband and friends of the lady, with the additional fact that, even if it were practicable to deliver by any other means, or were nature able to effect the delivery, the child could not possibly survive long, even if it were desirable that it should. They consented to the use of any means which we thought necessary. I immediately proceeded with the perforating scissors to open the head, which gave exit to an enormous quantity of a semi-pellucid fluid. As it was discharged while the patient lay on her back in the bed the exact amount could not be ascertained, but I am sure it may be safely estimated at a gallon. The most of it being evacuated the head collapsed, and two or three pains effected the delivery.

The child was of about the ordinary size, judging from its arms, legs, neck, and face. The hydropic accumulation was accompanied by extensive spina-bifida, and an absence of three of the lower lumbar vertebra, and I am certain there was a perfect and free intercommunication between the cranial, thoracic, and abdominal cavities, as the thorax and abdomen presented the appearance of having been evacuated. The child evinced some signs of vitality after its delivery by slight movements of the arms and legs, but it did not cry. The circumstances surrounding this case did not admit of an examination of the internal organs, which we very much regretted.

The above case is not reported on account of any supposed novelty in the treatment of it, but on account of the comparatively rare occurrence of such instances. Since it occurred I have read with interest the case reported in this Journal by Dr. Prentis, in his account of which he alludes to an analogous case reported in a late number of the Boston Medical and Surgical Journal. Our patient has entirely recovered.

On a review of the case, I am persuaded that the operation was indispensable to its safe termination. The uterine contractions were powerful, but availed nothing towards the expulsion of the fetus. I am confident that it could not have been born without the reduction of the head.

Shelbyville, May 11, 1849.

